# Digital therapeutics use burden in patients with chronic diseases: a scoping review

**DOI:** 10.3389/fpubh.2026.1919598

**Published:** 2026-08-12

**Authors:** Xinyu Feng, Xiangshu Cui

**Affiliations:** School of Nursing, Yanbian University, Yanji, China

**Keywords:** chronic diseases, digital health, digital therapeutics, DTx use burden, scoping review

## Abstract

**Objective:**

To systematically map the available evidence on the potential use burden of digital therapeutics (DTx) and DTx-related therapeutic digital interventions in patients with chronic diseases and to inform clinical implementation and policy evaluation.

**Methods:**

PubMed, Web of Science (WOS), Cochrane Library, Embase, Wiley, Scopus, CNKI, WanFang Data, VIP, and SinoMed databases were searched from inception to April 28, 2026. Studies were screened according to the inclusion and exclusion criteria. Data were extracted on application areas, potential sources of DTx use burden, indirect burden-related evaluation indicators, and support strategies potentially relevant to reducing DTx use burden.

**Results:**

A total of 11 studies were included. DTx and DTx-related therapeutic digital interventions for patients with chronic diseases were mainly applied in diabetes, hypertension, coronary heart disease, chronic insomnia, cancer treatment support, and inflammatory bowel disease. None of the included studies used a dedicated instrument to directly measure DTx use burden. Instead, burden-related evidence was mainly derived from indirect indicators, including utilization and participation indicators, module and task completion indicators, data recording indicators, acceptability and satisfaction indicators, and technical problems and safety records. The task requirements, use difficulties, and indirect indicators reported in the included studies suggested five potential sources of DTx use burden: digital access burden, data recording burden, task completion burden, continuous use burden, and technical operation burden.

**Conclusion:**

Evidence on DTx use burden in patients with chronic diseases remains preliminary, and direct measurement of patients’ subjective DTx use burden is limited. Future studies should combine patient-reported DTx use burden with objective usage data and documented reasons for discontinuation, and incorporate burden-related outcomes into implementation and policy evaluation to improve accessibility, continuity of use, equity, and implementation sustainability.

## 1 Introduction

The burden of chronic diseases continues to increase, and non-communicable diseases have become a leading cause of death worldwide, accounting for 75% of global non-pandemic-related deaths (1). Patients with chronic diseases are required to undertake long-term tasks, including medication management, health monitoring, follow-up visits, lifestyle adjustment, and symptom management. These ongoing disease-management tasks already impose a substantial treatment burden. Treatment burden theory suggests that when the workload required for treatment and self-management exceeds patients’ capacity, adherence and health outcomes may be adversely affected (2).

Digital therapeutics (DTx) are software-based interventions designed to deliver evidence-based therapeutic content for the prevention, management, or treatment of health conditions and have become increasingly relevant to chronic disease management (3). However, while DTx may improve the continuity of disease management, they may also shift additional digital tasks to patients, including technical operation, task learning, data recording, and continuous interaction. For older adults, patients with low digital literacy, or those with limited access to digital devices, these digital requirements may further increase DTx use burden. Digital health literacy research emphasizes that patients need the ability to access, understand, evaluate, and apply health information before using electronic health resources (4). The World Health Organization (WHO) has also emphasized that digital health development should prioritize accessibility, equity, and integration into health systems (5). Previous reviews have examined treatment burden in relation to telehealth, the cumulative burden associated with the concurrent use of multiple digital health technologies in patients with multimorbidity, and the broader effects of digital interventions on treatment burden in people with chronic conditions (6–8). However, these reviews did not specifically distinguish DTx use burden from overall treatment burden or systematically map the patient work and use difficulties associated with DTx-related interventions. The present review therefore focuses on distinguishing potential sources of DTx use burden from indirect burden-related evaluation indicators and mapping both across chronic disease groups.

Therefore, systematically mapping the potential sources of DTx use burden, indirect burden-related evaluation indicators, and reported support strategies is important for optimizing clinical implementation, supporting sustained use, and informing policy evaluation of DTx.

## 2 Methods

This scoping review was conducted according to the methodological framework proposed by Arksey et al., and was reported in accordance with the Preferred Reporting Items for Systematic Reviews and Meta-Analyses Extension for Scoping Reviews (PRISMA-ScR) (9). The study protocol was registered on the Open Science Framework (OSF) platform (10.17605/OSF.IO/JRE6T).

### 2.1 Research questions

The review addressed the following research questions: (1) Which disease groups and application scenarios are represented in studies reporting potential DTx use burden or burden-related information associated with DTx and DTx-related therapeutic digital interventions? (2) What potential sources and specific manifestations of DTx use burden have been reported in the included studies? (3) What indirect burden-related evaluation indicators and measurement methods have been used to describe DTx use and potential burden-related experiences? (4) What support strategies potentially relevant to reducing DTx use burden have been reported or implemented?

### 2.2 Literature search strategy

A comprehensive literature search was conducted in PubMed, Web of Science (WOS), Cochrane Library, Embase, Wiley, Scopus, CNKI, WanFang Data, VIP, and SinoMed databases. The search period was from database inception to April 28, 2026. Subject headings and free-text terms were combined in the search strategy. In addition, the reference lists of the included studies were manually searched to identify potentially eligible studies that may have been missed.

Because few existing studies explicitly reported DTx use burden, burden-related information was also included in the search strategy to improve retrieval sensitivity. The search terms included terms related to chronic diseases, such as “chronic disease,” “heart failure,” “hypertension,” “coronary disease,” “stroke,” “metabolic syndrome,” “diabetes mellitus,” “cancer,” “obesity,” “neoplasms,” and “chronic obstructive pulmonary disease”; terms related to DTx or DTx-related therapeutic digital interventions, such as “digital therapeutics,” “DTx,” “prescription digital therapeutic,” “digital behavioral therapeutic,” and “digital therapeutic intervention”; and terms related to use burden or burden-related information, such as “digital burden,” “technology burden,” “self-management burden,” “burden of treatment,” “usability,” “acceptability,” “feasibility,” “engagement,” “adherence,” “compliance,” “completion,” and “satisfaction.” The PubMed search strategy is shown in Table 1.

**Table 1:** Search strategy.

Step	Search formulation	Number
#1	“Chronic Disease”[Mesh] OR “Heart Failure”[Mesh] OR “Hypertension”[Mesh] OR “Coronary Disease”[Mesh] OR “Stroke”[Mesh] OR “Metabolic Syndrome”[Mesh] OR “Diabetes Mellitus”[Mesh] OR “Neoplasms”[Mesh] OR “Obesity”[Mesh] OR “Pulmonary Disease, Chronic Obstructive”[Mesh] OR “chronic disease”[Title/Abstract] OR “heart failure”[Title/Abstract] OR hypertension[Title/Abstract] OR “coronary disease”[Title/Abstract] OR stroke[Title/Abstract] OR “metabolic syndrome”[Title/Abstract] OR “diabetes mellitus”[Title/Abstract] OR cancer[Title/Abstract] OR obesity[Title/Abstract] OR neoplasms[Title/Abstract] OR “chronic obstructive pulmonary disease”[Title/Abstract] OR COPD[Title/Abstract]	7,928,897
#2	“digital therapeutics”[Title/Abstract] OR DTx[Title/Abstract] OR “prescription digital therapeutic”[Title/Abstract] OR “digital behavioral therapeutic”[Title/Abstract] OR “digital therapeutic intervention”[Title/Abstract]	3,212
#3	“digital burden”[Title/Abstract] OR “technology burden”[Title/Abstract] OR “self-management burden”[Title/Abstract] OR “burden of treatment”[Title/Abstract] OR usability[Title/Abstract] OR acceptability[Title/Abstract] OR feasibility[Title/Abstract] OR engagement[Title/Abstract] OR adherence[Title/Abstract] OR compliance[Title/Abstract] OR completion[Title/Abstract] OR satisfaction[Title/Abstract]	1,198,328
#4	#1 AND #2 AND #3	138

### 2.3 Literature inclusion and exclusion criteria

The inclusion and exclusion criteria were formulated according to the Population, Concept, and Context (PCC) framework (10). The inclusion criteria were as follows: (1) Population (P) were patients with chronic diseases, including but not limited to hypertension, diabetes, cancer, coronary heart disease, chronic insomnia, and inflammatory bowel disease; (2) Concept (C) involved DTx and DTx-related therapeutic digital interventions with explicit therapeutic or disease-management purposes, such as mobile apps, web-based platforms, digital behavioral interventions, and remote support tools with clear disease-management goals. For the purpose of this review, eligible DTx-related interventions were operationally defined as software-based digital interventions with explicit therapeutic or disease-management purposes that were used for the prevention, treatment, rehabilitation, or long-term management of chronic diseases and evaluated as the primary intervention in the included studies. The included studies also had to contain burden-related information reported or generated during patients’ use of the intervention. Burden-related information included directly measured DTx use burden or patient workload associated with DTx use, as well as indirect indicators that could reflect potential DTx use difficulties, such as technical problems, acceptability, feasibility, participation, adherence, compliance, completion, and satisfaction; (3) Context (C) included the prevention, treatment, rehabilitation, or long-term management of chronic diseases.

The exclusion criteria were as follows: (1) full text could not be obtained; (2) duplicate publications; and (3) reviews, meta-analyses, conference abstracts, dissertations, case reviews, expert reviews, and other non-original studies.

### 2.4 Literature screening and data extraction

All retrieved records were imported into NoteExpress for duplicate removal. Two reviewers independently screened titles and abstracts and subsequently assessed full-text articles against the eligibility criteria. Disagreements mainly concerned whether an intervention met the operational definition of DTx-related therapeutic digital interventions and whether the study reported information relevant to potential DTx use burden. All disagreements were resolved through discussion between the two reviewers; third-reviewer adjudication was not required. For descriptive presentation, the indirect burden-related evaluation indicators reported in the included studies were organized according to the aspect of DTx use that they represented.

Two researchers independently extracted data using a standardized data extraction form and cross-checked the extracted information. The extracted information included author, year of publication, country, study design, study population, sample size, application area, potential sources of DTx use burden, indirect burden-related evaluation indicators and measurement methods, and support strategies potentially relevant to reducing DTx use burden.

## 3 Results

The findings were descriptively synthesized in terms of DTx application areas, potential sources of DTx use burden, indirect burden-related evaluation indicators, and support strategies potentially relevant to reducing DTx use burden. None of the 11 included studies used DTx use burden as an independent primary outcome or applied a dedicated instrument to directly measure patients’ subjective DTx use burden. Accordingly, the findings should be interpreted as burden-related tasks, use difficulties, and indirect signals during DTx use rather than direct evidence of patients’ subjective DTx use burden.

### 3.1 Study selection

**Figure 1:**
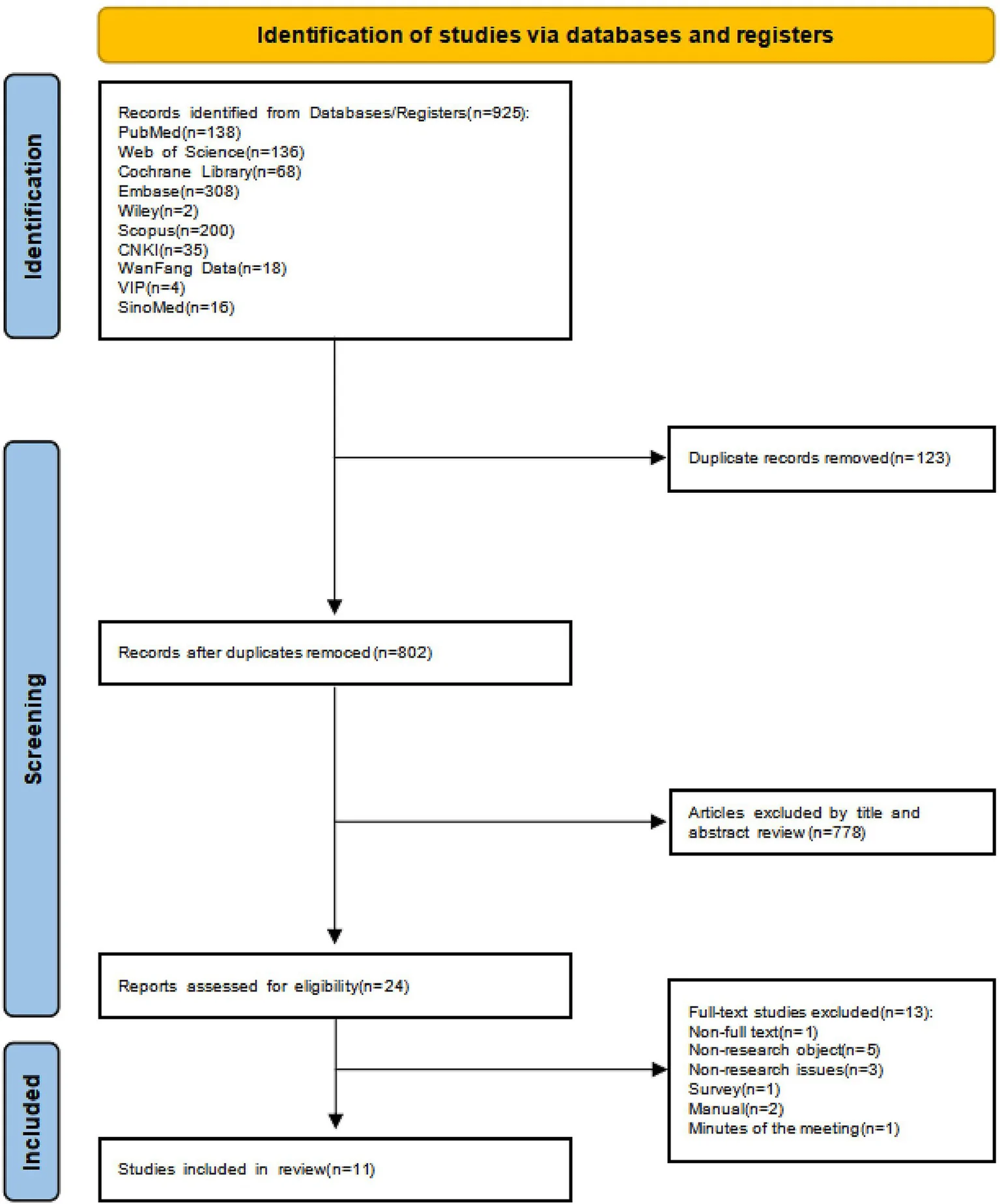
PRISMA flow diagram.

The initial database search identified 925 records. After duplicate removal and screening according to the inclusion and exclusion criteria, 11 studies were finally included in this review (11–21). The study selection process is presented in Figure 1.

### 3.2 Study characteristics

A total of 11 studies were included. The basic information of the included studies is shown in Table 2.

**Table 2:** Basic information of the included studies.

Author (s)	Year	Country	Study type	Study population	Sample size	Application area
Berman et al. (11)	2018	United States	Non-experimental study	Patients with type 2 diabetes mellitus	118	Diet, exercise, and self-management of blood glucose
Chawla et al. (12)	2022	India	Experimental study	Patients with type 2 diabetes mellitus	128	Blood glucose management and lifestyle management
Hommel et al. (13)	2023	United States	Experimental study	Adolescents with inflammatory bowel disease	22	Identification of self-management barriers and individualized interventions
Kario et al. (14)	2021	Japan	Experimental study	Patients with essential hypertension	146	Home blood pressure monitoring and lifestyle interventions
Thorndike et al. (15)	2024	United States	Non-experimental study	Patients with chronic insomnia	1,565	Digital cognitive behavioral therapy and sleep management
Li et al. (16)	2022	China	Experimental study	Patients with coronary heart disease	290	Post-discharge home management and secondary preventive medication management
Kario et al. (17)	2021	Japan	Experimental study	Patients with essential hypertension	390	Non-pharmacological treatment for hypertension and lifestyle interventions
Krishnakumar et al. (18)	2021	India	Non-experimental study	Patients with type 2 diabetes mellitus	102	Blood glucose control and diabetes self-management
Gudmundsson et al. (19)	2022	Iceland	Experimental study	Cancer patients	29	Lifestyle, symptom support, and PRO monitoring during cancer treatment
Hsia et al. (20)	2022	United States	Experimental study	Patients with type 2 diabetes mellitus	669	Digital behavioral therapy and blood glucose management
Ritterband et al. (21)	2022	United States	Non-experimental study	Patients with chronic insomnia	7,216	Digital cognitive behavioral therapy and sleep management

### 3.3 Disease groups and application areas of DTx

The included studies showed that DTx and DTx-related therapeutic digital interventions were mainly applied in type 2 diabetes (11, 12, 18, 20), essential hypertension (14, 17), coronary heart disease (16), chronic insomnia (15, 21), cancer treatment support (19), and adolescent inflammatory bowel disease (13), with type 2 diabetes being the most frequently studied disease group. These DTx-related interventions were mainly delivered through mobile applications, web-based platforms, or prescription digital therapeutics. Their application areas included health education, self-monitoring, data recording, behavioral intervention, reminder feedback, individualized program formulation, and remote support.

Specifically, interventions for type 2 diabetes mainly focused on diet, exercise, blood glucose, weight, and self-care behavior management (11, 12, 18, 20). Interventions for essential hypertension were mainly combined with home blood pressure monitoring and lifestyle intervention (14, 17). The intervention for coronary heart disease focused on post-discharge home management and secondary prevention medication compliance (16). Interventions for chronic insomnia mainly involved digital cognitive behavioral therapy and sleep diary completion (15, 21). The intervention for cancer treatment support was mainly used for symptom support, lifestyle guidance, and patient-reported outcome (PRO) monitoring (19). The intervention for adolescent inflammatory bowel disease focused on self-management barrier identification and individualized intervention (13).

### 3.4 Types of DTx use burden in patients with chronic diseases

Across the 11 studies of DTx and DTx-related therapeutic digital interventions, the reported task requirements and use difficulties were descriptively organized into five potential sources of DTx use burden: digital access burden, data recording burden, task completion burden, continuous use burden, and technical operation burden.

First, digital access burden was mainly reflected in requirements for device ownership, language ability, and digital operational skills. Berman et al. (11) required participants to own an Android or iPhone device and excluded those who were unable to read or write in English or had insufficient computer literacy to operate the app. Chawla et al. (12) required participants to have functional English reading ability and access to a smartphone capable of running the intervention app. Li et al. (16) required Chinese reading ability and excluded participants who were still unable to operate the mobile app after training. In the hypertension study by Kario et al. (14), participants were required to use a smartphone daily, and in the HERB Digital Hypertension 1 (HERB-DH1) study (17), some patients were excluded because they did not have an appropriate smartphone or were unable to use the smartphone app.

Second, data recording burden was mainly associated with the continuous recording or uploading of health-related data, including diet, weight, physical activity, blood glucose, blood pressure, heart rate, symptoms, sleep diary, and patient-reported outcomes. For example, Krishnakumar et al. (18) required participants to record diet, weight, physical activity, and blood glucose simultaneously. The HERB system in the study by Kario et al. (14) required participants to upload home blood pressure and daily activity data. Ritterband et al. (21) and Thorndike et al. (15) required sleep diary completion, while Gudmundsson et al. (19) required participants to record steps, diet, drinking water, and PRO data.

Third, task completion burden was mainly related to the need to complete educational courses, behavioral modules, core therapeutic modules, or daily tasks. For example, BT-001 required participants to complete treatment modules (20), and HERB-DH1 required participants to complete Step 1 and Step 2 recommendations (17). Somryst (15) and SHUTi (21) both included six core therapeutic modules, while the Self-Management Assistance for Recommended Treatment (SMART) portal included 16 intervention modules, with each module lasting approximately 20 min (13).

Fourth, continuous use burden was reflected in the need to maintain frequent app use, task interaction, or module completion over time. Berman et al. (11) reported that participants interacted with the app an average of 4.3 times per day. In the pilot hypertension study by Kario et al. (14), app use decreased from 100% at weeks 12 and 16 to 57% at week 24, and the 24-week app use rate was lower among patients aged ≥65 years than among those aged <65 years. In the Somryst study (15), only 58.0% of participants completed four core modules, and 46.8% completed all six core modules. Cancer DTx users completed an average of 7.7 task interactions per day (19). Hommel et al. (13) also reported lower-than-expected parent engagement and higher-than-expected attrition.

Fifth, technical operation burden was mainly reflected in device connection, data synchronization, and system operation problems. Chawla et al. (12) involved connections between the app and Bluetooth-enabled blood glucose meters, blood pressure monitors, and fitness applications. In the pilot hypertension study by Kario et al. (14), participants used a home blood pressure monitoring device connected to the app via Bluetooth. The HERB-DH1 study (17) reported temporary system failures in 48 patients, including Bluetooth connection failure, abnormal app content display, inability to enter data, and inability to start the app.

### 3.5 Evaluation indicators and measurement methods of DTx use burden

Because none of the included studies used a dedicated instrument to directly measure DTx use burden, the burden-related evaluation indicators were descriptively organized according to the aspect of DTx use documented in the original studies. Indicators describing app uptake, retention, frequency of use, or program engagement were summarized as utilization and participation indicators. Indicators describing the completion of prescribed therapeutic modules, educational content, or intervention tasks were summarized as module and task completion indicators. Indicators describing patient-entered or uploaded health information were summarized as data recording indicators. User-reported perceptions of feasibility, usefulness, acceptability, or satisfaction were summarized as acceptability and satisfaction indicators. Reported system failures, device-related problems, adverse events, or safety information were summarized as technical problems and safety records. These categories were used to facilitate the descriptive presentation of heterogeneous evidence and should not be interpreted as validated measures of patients’ subjective DTx use burden.

First, utilization and participation indicators included app retention rate, frequency of app interaction, program participation, and app use rate. Berman et al. (11) reported that 86.2% of participants were still using the app at week 12, with a mean of 4.3 app interactions per day. Krishnakumar et al. (18) analyzed intervention effects according to program participation, while Chawla et al. (12) classified program participation into low, medium, and high levels. In the pilot hypertension study, Kario et al. (14) calculated the app use rate based on the number of days on which participants launched the app within 7 days before each visit. In the HERB-DH1 study, mobile app participation rate and app-guided behavioral execution score were used to evaluate intervention use (17).

Second, module and task completion indicators included the number of completed treatment modules, completion rates of core programs, daily task interactions, and completion of intervention modules. Hsia et al. (20) used the number of completed BT-001 modules to reflect intervention exposure, with participants completing a mean of eight modules within 90 days. The Somryst study reported the completion rate of four core modules and the completion rate of all six core modules (15). Ritterband et al. (21) used the number of completed core modules and sleep diary entries to indicate program use. Gudmundsson et al. (19) reported program completion rate, active days, and daily task interactions. Hommel et al. (13) reported SMART portal module use and the participation of patients and parents.

Third, data recording indicators included records of blood glucose, blood pressure, diet, weight, physical activity, sleep diary, and patient-reported outcomes. Krishnakumar et al. (18) collected diet, weight, physical activity, and blood glucose data recorded by participants in the app. Hsia et al. (20) reported the recording frequency of physical activity, plant-based diet, and fasting blood glucose. Kario et al. (17) collected home blood pressure data, app use data, and educational program progress. Li et al. (16) used the proportion of guideline-recommended secondary prevention medications at 12 months as the primary outcome, and also recorded medication compliance, blood pressure control, and lipid control at 6 months. Ritterband et al. (21) recorded sleep diary completion, while Gudmundsson et al. (19) collected patient-reported outcomes, step-goal completion, and PRO records in the app.

Fourth, acceptability and satisfaction indicators included user feedback and satisfaction-related evaluation. Gudmundsson et al. (19) used a satisfaction questionnaire to assess willingness to recommend the intervention, perceived support for coping with disease, and perceived usefulness of medication reminders. Hommel et al. (13) evaluated the feasibility and acceptability of the SMART portal through user feedback.

Fifth, technical problems and safety records included temporary system failures, device-related problems, adverse events, and safety information. Kario et al. (17) recorded temporary system failures, including Bluetooth connection failure, abnormal app content display, inability to enter data, and inability to start the app, as well as adverse events. The pilot hypertension study by Kario et al. (14) and the study by Hsia et al. (20) mainly reported adverse events or device-related safety outcomes.

Overall, the potential sources of DTx use burden and indirect burden-related evaluation indicators differed across disease groups. The matrix of DTx use burden types and evaluation indicators across disease groups is presented in Table 3.

**Table 3:** Matrix of DTx use burden types and evaluation indicators across disease groups.

Disease group	Included studies	Digital access burden	Data recording burden	Task completion burden	Continuous use burden	Technical operation burden	Utilization and participation indicators	Module and task completion indicators	Data recording indicators	Acceptability and satisfaction indicators	Technical problems and safety records
Type 2 diabetes	Berman et al. (11); Chawla et al. (12); Krishnakumar et al. (18); Hsia et al. (20)	●	●	●	●	○	●	●	●	○	○
Essential hypertension	Kario et al. (14); Kario et al. (17)	●	●	●	●	●	●	●	●	—	●
Coronary heart disease	Li et al. (16)	●	●	○	○	—	○	—	●	—	—
Chronic insomnia	Thorndike et al. (15); Ritterband et al. (21)	—	●	●	●	—	○	●	●	—	—
Cancer	Gudmundsson et al. (19)	—	●	●	●	—	●	●	●	●	—
Adolescent inflammatory bowel disease	Hommel et al. (13)	—	—	●	●	—	●	●	—	●	—

● indicates clearly involved; ○ indicates partially or indirectly involved; — indicates not explicitly reported. The matrix was summarized based on burden-related information reported in the included studies. The findings mainly reflect potential sources of DTx use burden and burden-related indicators, rather than direct measurements of patients’ subjective DTx use burden.

### 3.6 Support strategies to reduce DTx use burden

The support strategies reported in the included studies were mainly implemented before and during DTx use and throughout follow-up. Although these strategies may be relevant to reducing DTx use burden, none of the included studies directly evaluated burden reduction as a predefined outcome.

Before use, the strategies mainly focused on operational preparation and intervention development. Li et al. provided operational training before intervention initiation (16). Hommel et al. (13) involved patients, parents, and multidisciplinary healthcare professionals in the development of the SMART portal to improve its alignment with patients’ self-management needs.

During use, the strategies mainly relied on automated or professional support to facilitate the completion of intervention-related tasks. These strategies included telephone guidance from health coaches (11), artificial intelligence (AI) chatbot feedback (18), telephone or chat support from certified diabetes educators (18), digital consultation with doctors, nutritionists, or sports coaches (12), individualized advice from virtual nurses (17), and weekly support from exercise physiologists (19).

During follow-up, the strategies included data sharing, remote monitoring, and continued professional support. In the coronary heart disease study, the DTx shared patients’ home blood pressure, heart rate, symptoms, and medication-related information with doctors and nurses for follow-up and program adjustment (16). In the hypertension study, the DTx connected home blood pressure monitoring with healthcare professional management through a patient-facing app, a cloud server, and a clinician-facing web-based management platform (14). The SMART portal developed by Hommel et al. (13) provided self-management support for patients, parents, and clinicians.

## 4 Discussion

One important finding of this review is that DTx use burden in patients with chronic diseases has not been sufficiently measured directly. Although the included studies covered several disease fields, including diabetes, hypertension, coronary heart disease, chronic insomnia, cancer treatment support, and inflammatory bowel disease, their primary focus was clinical effectiveness, feasibility, utilization and participation indicators, module and task completion indicators, and technical problems and safety records. Patients’ subjective DTx use burden has not yet been systematically evaluated as an independent outcome. Therefore, the five potential sources summarized in this review were derived from task requirements and use difficulties during DTx use and should not be interpreted as directly measured or fully quantified DTx use burden.

The available evidence was unevenly distributed across disease groups, with a relatively greater concentration of studies in type 2 diabetes and essential hypertension. Nevertheless, the five potential sources of DTx use burden identified in this review—digital access burden, data recording burden, task completion burden, continuous use burden, and technical operation burden—represent recurring requirements of DTx-supported chronic disease management and may also be relevant across different chronic disease populations. However, their specific manifestations and relative importance may vary according to disease trajectory, symptom profile, treatment complexity, monitoring requirements, and DTx use context. Therefore, the findings should be regarded as preliminary evidence for understanding potential DTx use burden across chronic disease populations, rather than as a definitive classification applicable to all chronic diseases.

### 4.1 Patient-reported DTx use burden should be considered alongside clinical effectiveness

The results showed that existing studies have not yet used dedicated instruments to evaluate DTx use burden. Preliminary progress has been made in the development (22) and validation (23) of patient-reported measures of the burden of digital care, a construct closely related to DTx use burden. These developments suggest growing attention to the patient workload associated with digital care. However, such measures have not yet been widely applied in studies of DTx for chronic disease management. Future research should use or adapt patient-reported measures relevant to DTx use burden, including digital care-specific measures such as the Treatment Burden Questionnaire Plus Digital (TBQ+D), to capture the workload and burden experienced during actual use (24).

At the same time, DTx use burden may encompass the workload experienced by patients when using DTx for disease management, including task demands, time investment, adjustments to daily life, and psychological pressure. When treatment tasks exceed patients’ capacity, adherence and health outcomes may be affected (2). Therefore, DTx evaluation should not focus only on clinical efficacy, but should also take into account the time, energy, and operational costs required for patients to complete digital tasks.

Current evaluation and reporting frameworks for digital health technologies have addressed usability, remote data acquisition, implementation processes, and reporting quality, such as the National Institute for Health and Care Excellence (NICE) digital health technology evidence standard and the Consolidated Standards of Reporting Trials of Electronic and Mobile Health Applications and Online Telehealth (CONSORT-EHEALTH) (25–27). However, based on the findings of this review, DTx use burden is still mainly identified indirectly after implementation. Digital access burden, data recording burden, task completion burden, continuous use burden, and technical operation burden should be considered earlier in DTx design and testing as important components of patient experience and implementation sustainability. Future DTx developers and researchers should assess the anticipated interaction frequency, data-entry requirements, and technical support needs during intervention design and pilot testing while ensuring clinical efficacy and safety, and reduce potential use burden by simplifying operation procedures, enhancing automated data collection, and providing timely support.

### 4.2 Continuous digital tasks may constitute important sources of DTx use burden

DTx use burden was mainly reflected in digital access burden, data recording burden, task completion burden, continuous use burden, and technical operation burden. Patients with chronic diseases already need to undertake long-term medication, symptom monitoring, follow-up visits, and lifestyle adjustments. DTx-related interventions may additionally require patients to complete digital tasks related to blood glucose, blood pressure, diet, exercise, sleep diary, patient-reported outcomes, and treatment modules, which may increase patients’ workload beyond routine disease management.

However, existing studies have not measured patients’ subjective burden associated with these tasks. Therefore, whether continuous digital tasks are truly transformed into perceived use burden, and the extent of such burden, still require further verification in future studies. Both treatment burden theory and minimally disruptive medicine emphasize that chronic disease management should minimize interference with patients’ daily lives while achieving therapeutic goals, and should avoid transferring excessive management responsibility to patients (28). Therefore, future DTx development and evaluation should pay greater attention to task frequency, module duration, data recording complexity, and feedback mode, reduce unnecessary repetitive input and excessive reminders, and enable DTx to serve as a supportive management tool rather than a new source of use burden.

### 4.3 Attention should be paid to use difficulties among older adults and patients with low digital literacy

In the included studies, some DTx required patients to have smartphones, language reading ability, and basic digital operational skills. This suggests that existing studies may have preferentially included patients with better digital resources and stronger operational capacity. These requirements are closely related to digital access burden and technical operation burden, and may affect patients’ ability to initiate and continuously use DTx.

Digital health literacy refers to the ability of individuals to access, understand, evaluate, and apply electronic health information. The eHealth Literacy Scale (eHEALS) developed by Norman and Skinner assesses individuals’ perceived skills in finding, evaluating, and applying electronic health information (4). For older adults, patients with low education levels, low income, or limited access to digital devices, digital health services may further create new forms of health inequality. Recent research on the digital divide among older adults has also shown that the digital divide is associated with factors such as education, income, race, and access to health resources, and may influence older adults’ access to digital health services (29).

Therefore, DTx research should pay attention to digitally disadvantaged populations in inclusion criteria, intervention design, and implementation. This may help reduce patient selection bias and differences in service accessibility caused by device requirements, language ability, and operational thresholds.

### 4.4 DTx use burden should be considered in implementation and policy evaluation

App use, participation, module completion, and data recording may indicate difficulties during DTx use, but they cannot independently establish patients’ subjective DTx use burden. Digital engagement is influenced by perceived benefit, intervention design, disease status, motivation, and competing demands, while nonuse and study withdrawal may represent different forms of attrition (30, 31). Future studies should therefore combine patient-reported DTx use burden with objective usage data and documented reasons for discontinuation. At minimum, evaluations should report time spent on digital tasks, frequency of manual data entry, technical support contacts, and changes in burden over time.

DTx use burden should also be evaluated in relation to implementation context. Operational guidance, automated feedback, professional consultation, and remote monitoring may reduce task difficulty, but the included studies did not directly test whether these measures reduced patient-reported burden. Implementation studies should compare different levels of support and determine whether their effects justify the additional personnel, technical, and organizational resources required (32, 33). Because burden may shift from initial learning and technical operation to repetitive recording and sustained task completion, assessment should be conducted at multiple stages rather than only at the end of an intervention. At the service-delivery level, prescription DTx should be integrated into existing primary chronic disease management pathways, with clear responsibilities for patient eligibility assessment, technical support, review of patient-generated data, and follow-up when use difficulties occur.

International prescription DTx policies further increase the need for such evidence. Germany’s Digital Health Applications (DiGA) pathway and France’s Advance Digital Care (PECAN) early-coverage mechanism, and Medicare payment for selected digital mental health treatment devices in the United States illustrate the expansion of regulatory and reimbursement routes for digital treatments (34–36). In addition to clinical effectiveness and safety, health technology assessment and real-world evaluation should examine patient-reported DTx use burden, discontinuation, technical support requirements, accessibility, equity, and sustained use. The five potential sources identified in this review may inform these evaluations, but should not be treated as a validated assessment framework. Linking reimbursement continuation and scale-up decisions to patient-level implementation evidence may reduce the risk that expanded access transfers excessive treatment work to patients.

## 5 Limitations

This scoping review systematically mapped DTx use burden in patients with chronic diseases according to the Arksey et al. framework and PRISMA-ScR. Several limitations should be acknowledged. First, only peer-reviewed original studies published in Chinese or English were included; relevant studies in other languages or the grey literature may have been missed. Second, only 11 studies were included, and the evidence was concentrated in type 2 diabetes and essential hypertension, limiting applicability to chronic disease populations with different treatment demands and digital care contexts. Third, no included study used a dedicated instrument to assess DTx use burden. The indirect burden-related evaluation indicators may reflect use difficulties, but may also be influenced by motivation, disease severity, perceived benefit, digital literacy, technical usability, and implementation context; they therefore cannot be equated with patients’ subjective DTx use burden. Finally, the reported support strategies were components of intervention delivery, and their effects on patient-reported DTx use burden were not directly evaluated.

## 6 Conclusion

This scoping review mapped the application areas, five potential sources of DTx use burden, indirect burden-related evaluation indicators, and reported support strategies in chronic disease management. None of the included studies directly measured patient-reported DTx use burden; app use, participation, module and task completion, data recording, acceptability, satisfaction, and technical problems therefore represent indirect signals rather than direct measurements. Digital access burden, data recording burden, task completion burden, continuous use burden, and technical operation burden should be interpreted as potential sources of DTx use burden.

Future studies should combine patient-reported DTx use burden with objective usage data, documented reasons for discontinuation, and repeated assessment across the intervention period. Support strategies should be evaluated for their effects on patient workload and sustained use, together with their personnel, technical, and organizational resource requirements. Health technology assessment, real-world evaluation, reimbursement continuation, and scale-up decisions should consider accessibility, equity, technical support needs, and implementation sustainability alongside clinical effectiveness and safety, with particular attention to older adults and people with low digital literacy or limited access to digital devices.
